# Supplementary material for: Effect of concentric exercise-induced fatigue on proprioception, motor control and performance of the upper limb in handball players
Source: PLoS One. 2024 Dec 26;19(12):e0315103. doi: 10.1371/journal.pone.0315103 (PMC11671013; doi:10.1371/journal.pone.0315103)
Supplement: Attachment — Submitted filename: Response to editor.docx [file pone.0315103.s001.docx]

**Response to editor**

1.Please provide non-author contact information (phone/email/hyperlink) for a data access committee, ethics committee, or other institutional body to which data requests may be sent. If applicable, please also provide any necessary information which interested researchers would need when requesting access to data in order to obtain the minimal data set for your study.

Directing data requests to a non-author institutional point of contact, such as a data access or ethics committee, helps guarantee long term stability and availability of data. Providing interested researchers with a durable point of contact ensures data will be accessible even if an author changes email addresses, institutions, or becomes unavailable to answer requests.

Answer:

I am writing you two personal emails, which will not change in the future:

1) [stelios.hadjisavvas@gmail.com](mailto:stelios.hadjisavvas@gmail.com)
2) [Manos.stefanakis.gr@gmail.com](mailto:Manos.stefanakis.gr@gmail.com)

Furthermore in the declaration section I have changed the data availability statement to this: “The data that supports the findings of this study are available in the Mendeley Data (Digital Commons Data Repository) at <https://data.mendeley.com/datasets/hrnyw4g89c/1>”
